# Attributing Farm-to-Slaughter Emissions to Hides: Evidence from Beef Supply Chains

**DOI:** 10.3390/ani15243546

**Published:** 2025-12-09

**Authors:** Mondina Francesca Lunesu, Fabio Correddu, Silvia Carta, Sara Sechi, Marco Farina, Giuseppe Pulina

**Affiliations:** Sezione di Scienze Zootecniche, Dipartimento di Agraria, University of Sassari, Viale Italia, 39, 07100 Sassari, Italy; mflunesu@uniss.it (M.F.L.); fcorreddu@uniss.it (F.C.); scarta2@uniss.it (S.C.); ssechi1@uniss.it (S.S.); m.farina8@phd.uniss.it (M.F.)

**Keywords:** leather, beef by-product, bovine hides, carbon footprint, life cycle assessment, allocation

## Abstract

To ensure transparent Life Cycle Assessment (LCA), climate impacts from cattle production must be consistently allocated among meat, milk and raw hides. This review examines allocation boundaries, compares physical and economic methods, and evaluates the upstream burden attributable to hides using extensive data from the Italian beef sector. Three hypotheses were tested: that hides, as marketable co-products, bear a non-zero share of upstream emissions (H1); that the burden assigned by economic allocation is lower than that assigned by physical (mass-based) allocation (H2); that allocation shares vary over time according to hide/meat price ratios (H3). The results from large-scale Italian datasets confirmed all three hypotheses. Physical allocation attributed an average of 5.9% of live weight to hides, whereas economic allocation assigned an average of 2.7% in 2023, decreasing over the historical price series. Consistent with available inventories, the upstream carbon footprint of raw hides was found to range from 1.63 kg CO_2_*e*/kg (economic allocation) to 3.55 kg CO_2_*e*/kg (physical allocation) when GWP_100_ was used. These findings demonstrate that neglecting co-product allocation leads to the systematic overestimation of the environmental impact of meat and the underestimation of the environmental impact of leather. Overall, economic allocation is the most appropriate and policy-aligned approach to hide accounting, reflecting market value and reducing methodological bias in beef-chain life cycle assessments (LCAs).

## Simple Summary

The environmental impact of cattle farming is typically only attributed to meat or milk production, overlooking the valuable co-product of raw hides entering the leather supply chain. Failing to assign an upstream impact to them produces biased estimates of the climate burden of beef and undervalues the role of co-product recovery in circular economy systems. This review explains why raw hides should not be treated as waste, summarises the allocation methods commonly used in life cycle assessment (LCA) and presents Italian data showing the effect of different allocation approaches (mass-based, bio-physical and economic) on the share of emissions assigned to hides. The results confirm that hides bear a small but non-zero share of upstream emissions, that economic allocation generally assigns them the lowest share and that price fluctuations influence these values over time. Recognising the upstream environmental impact of hides provides a more accurate representation of cattle production systems and supports the transparent circular use of bovine resources.

## 1. Introduction

### 1.1. Livestock Emissions and Climate Metrics

Livestock production is widely considered one of the main sources of non-CO_2_ agricultural emissions; it accounts for two-thirds of these and thus represents 7.4% of global emissions, excluding land use, land use change and forestry (LULUCF) [1].

The greenhouse gas (GHG) emissions are measured, in accordance with international standards (ISO 14060 series), using carbon dioxide equivalents (CO_2_*e*), which translates the climate impact of the various greenhouse gases into CO_2_ terms. This equivalence is known as the Global Warming Potential (GWP) and evaluates how much a given GHG contributes to global warming compared with CO_2_. According to this standard equivalence, over a 100-year period, the GWP_100_ of methane (CH_4_) is 28 times that of CO_2_ (1 kg CH_4_ = 28 kg CO_2_*e*), while for nitrous oxide (N_2_O), the GWP_100_ is 273 times that of CO_2_ (1 kg N_2_O = 273 kg CO_2_*e*) [2]. However, the different behaviour of these GHGs in the atmosphere (CO_2_ and N_2_O persist for centuries, whereas CH_4_ is removed within few decades) has led to the development of new metrics, the most widespread of which are the Global Temperature Potential (GTP) [2] and the Global Warming Potential star (GWP*, with values expressed as warming equivalent, *we*), the latter being the most used in the recent literature, and recognised by FAO [3] and IPCC [2], as it better matches the dynamics of temperature change induced in the atmosphere by climate-altering gases [4]. Specifically, over the standard GWP, which expresses the cumulative radiative forcing of a greenhouse gas over a specified time horizon (usually 100 years), other metrics have been developed to more accurately reflect the varying atmospheric behaviours of gases that alter the climate. The GTP quantifies the change in global mean surface temperature at a given future point in time following a pulse emission of a gas. This emphasises the long-term temperature response rather than cumulative forcing. While GWP_100_ and, partially, GTP are both suitable for comparing long-lived gases such as CO_2_ and N_2_O, they are less appropriate for short-lived climate pollutants such as CH_4_, which has an atmospheric lifetime of around ten years. For this reason, the most recent literature has increasingly adopted the GWP*, a metric designed specifically to capture the distinct temperature dynamics of short-lived gases. Unlike GWP_100_, which treats CH_4_ as if it accumulated in the atmosphere in the same way as CO_2_, GWP* relates changes in CH_4_ emissions to their actual warming effect. This recognises that stabilised CH_4_ emissions lead to a stable temperature impact, rather than an increasing one. The GWP*, therefore, provides a closer approximation of methane’s real contribution to global warming: rising emissions cause strong additional warming, stable emissions maintain existing warming and declining emissions generate a cooling effect. This behaviour is consistent with the physical properties of methane, which is removed from the atmosphere on decadal timescales. Explaining these differences also clarifies the contrast between the results reported under GWP and GWP* in Table 1. Under GWP_100_, methane is treated as a cumulative pollutant, resulting in a higher apparent contribution to warming. In contrast, under GWP*, the metric reflects the rate of change in emissions. This often results in substantially lower warming-equivalent values when emissions are declining or stable. This distinction is important for understanding methane dynamics in livestock systems and for comparing regions with different historical emission trends.

### 1.2. Methane Emissions from Livestock and European Trends

Methane represents the principal GHG emitted by livestock, which contributes to 32% of anthropogenic emissions of this gas. Consequently, methane is a crucial gas for understanding the climatic impact of farm animals. The Global Livestock Environmental Assessment Model (GLEAM), developed by the FAO [5], shows that methane from ruminants is a major contributor to GHG emissions, with enteric fermentation being the primary source. According to the FAOSTAT dataset, cattle (dairy + non-dairy) accounted for up to 72% of all enteric methane emissions in 2023 [6]. In addition, the FAO global assessment reports that approximately 90% of methane emissions from livestock originate from ruminants, with cattle representing the dominant source; specifically, dairy cattle emit the highest per-animal daily methane, while non-dairy (beef) cattle contribute the larger share of total livestock methane due to their greater numbers [3].

**Table 1:** Application of Global Warming Potential (GWP) and Global Warming Potential star (GWP*) to enteric methane emissions from 1990 to 2020 in Italy, the EU and the world.

	Emission (kt CH_4_) ^1^				Cumulative Impact (30 Years) ^2^			
	1990		2020		All Animals		Cattle	
	All Livestock	Cattle	All Livestock	Cattle	GWP (kt CO_2_*e*)	GWP* (kt CO_2_*we*)	GWP (kt CO_2_*e*)	GWP* (kt CO_2_*we*)
World	89,060	66,562	101,776	73,132	80,151,120	46,741,380	58,671,480	28,464,870
EU	8652	7436	6376	5519	6,311,760	−3,201,660	5,441,100	−2,665,425
Italy	793	674	580	477	576,660	−303,135	483,420	−292,845

^1^ Emission values from FAOSTAT [7]. ^2^ Cumulative impacts calculated for GWP_100_ and GWP* [8].

The European Union (EU-27) has been particularly successful in reducing GHG emissions from livestock systems, particularly enteric methane, compared to global trends. This progress reflects long-term structural changes in herd management, substantial increases in animal productivity, and improvements in feed quality and efficiency. It is also due to the adoption of more advanced manure management and regulatory frameworks across Member States [7].

Italy falls virtuously within the downward evolution of methane emissions from EU livestock. Global, European and Italian CH_4_ emissions, and relative 30-year cumulative impact expressed with standard (GWP_100_) and new metrics (GWP*), are reported in Table 1. Under the new metrics, for the EU and Italy, emissions of this gas show a negative contribution to atmospheric warming, while the global figure, especially for cattle, is more than halved.

### 1.3. Conceptual Framework and Study Objectives

For the sake of conceptual clarity and in view of the relevance of downstream material flows in the following sections, we adopt the following operational distinction, which is consistent with LCA practice: co-products are outputs that are intentionally obtained alongside the main product and that possess independent economic value; by-products are secondary outputs that are not primarily targeted by the production process, but which are still capable of partial economic valorisation; and waste comprises materials that bear only disposal costs and that do not generate revenue, including fractions that may eventually be used as low-value inputs in the feed, textile, pharmaceutical or fertiliser industries, or that may be diverted to anaerobic digestion for biogas production [9].

This study aims to assess whether raw bovine hides should bear a share of upstream GHG emissions from farming and slaughter, and how such allocation depends on methodological choices. Specifically, we test three hypotheses: H1, raw hides, being marketable co-products rather than waste, bear a non-zero share of upstream emissions; assigning a null footprint to raw hides [10] would artificially transfer the entire environmental burden of the upstream process to meat alone and this would distort the relative impact of co-products and violate standard LCA allocation principles, which require the burden to be distributed among all marketable outputs; H2, the burden assigned to hides under economic allocation is significantly lower than that under physical (mass-based) allocation, consistently with market valuation; H3, the hide share under economic allocation varies over time with the hide/meat price ratio; a multi-year average provides more stable estimates for Life Cycle Assessment (LCA) comparability.

Analyses were performed using Italian beef sector data provided by INALCA, the most important beef production hub in this Country, accounting for around 25% of all beef marketed nationally, and it is the only major company that publishes detailed environmental and production data through its publicly available Sustainability Report [11] and national price series, as detailed in the next section.

## 2. Materials and Methods

This review combines a descriptive analysis of the allocation methods used in cattle LCA with an empirical assessment based on Italian industrial data. The methodological approach comprised three consecutive phases.

First, a structured literature review was conducted to classify the allocation methods used for cattle products for distributing the environmental burdens, resource use, or inputs and outputs of the production system among multiple co-products (i.e., milk, meat, and slaughter by-products). In cattle production, allocation determines how impacts are divided between products such as milk, meat, and slaughter by-products, so that each product is assigned a fair share of the system’s total environmental load. This step drew on scientific articles, FAO technical reports, International Dairy Federation (IDF) guidance documents, and industrial LCA standards (e.g., PEFCR) to describe the rationale, assumptions, and limitations characterising bio-physical, physical, and economic allocation approaches.

Secondly, we used quantitative data from INALCA, Italy’s largest beef processing group, which handles around 750,000 slaughtered cattle per year. The INALCA was chosen because it represents a substantial share of the national beef market (approximately 25% of all beef sold in the country) and because it processes the full spectrum of slaughter categories, including young bulls, mixed lots, calves and cull cows. This provides a representative basis for comparing allocation outcomes across animal types. INALCA was also selected because it is the only major Italian operator that publishes detailed, regularly updated environmental and production information in its annual Sustainability Report [11]. This ensures that multi-year data on live weights, co-product yields and price series are publicly available, transparent and reproducible. Although other slaughterhouses operate in Italy, no comparable multi-year dataset with systematic disaggregation of co-products was accessible, making INALCA’s dataset uniquely suitable for rigorous allocation analysis.

Thirdly, the allocation shares were computed using physical allocation based on the proportion of hide weight to live weight, and economic allocation based on the ratio of hide to meat prices for each animal category. These results were then contextualised alongside the bio-physical allocation commonly adopted in dairy systems to facilitate a discussion of methodological coherence across cattle sectors. All GHG values refer to CO_2_*e* under GWP_100_ because implementing GWP* would require detailed temporal emission profiles for each slaughter category, and these are unavailable for the examined dataset.

## 3. Life Cycle Assessment and the Assessment of Climate-Altering Gas Emissions in Beef Supply Chains

The LCA is the most widely used method for quantifying emissions generated by livestock farms and is widely applied to cattle systems, encompassing the beef and dairy cattle and dairy buffalo sectors. In comparative analyses, it is also applied to small ruminants. Therefore, the choice of allocation method is critical, particularly when co-products are involved [12].

This method evaluates both GHG emissions and their impacts on the environment, on humans and on natural resource use over the entire production cycle of a good, service or process. The methodology considers all production stages, from raw-material extraction and transport to processing, distribution, use, re-use, recycling and final disposal (“cradle-to-grave” approach, ISO 14040:2006 and ISO 14044:2006) [13,14]. In this contest, the carbon footprint (CFP) can be defined as an LCA limited to a single impact category (climate change) and referred to a functional unit (kg of milk, body mass, protein, etc.). The CFP assesses the overall set of GHG emissions associated with a product, expressed in CO_2_*e* (or CO_2_*we* when using GWP*) [15]. A critical aspect for correct CFP calculation is defining the system boundary to include in the model and the allocation method for the overall impact among the different products, co-products and by-/sub-products of a production cycle. Although the ISO standards [13,14] discourage allocation, the case of bovine production is so specific that the scientific literature and environmental-audit professionals agree that the practice is unavoidable to correctly apportion impacts between milk and meat and, within the latter, between the main product and co-/sub-products [16]

Beef production emits GHGs along the entire chain (feed production, farming, processing and distribution). The LCA of beef evaluates the overall impact calculated as the system’s main output, using a functional unit (FU) represented by 1 kg of live weight, 1 kg of weight gain, 1 kg of carcass weight, or 1 kg of edible beef. The CFP generally ranges from 9 to 20 kg CO_2_*e* per kg of live weight. This variability is due to differences in production systems (intensive versus extensive), feed composition and quality, slaughter age, animal efficiency, and geographic and climatic conditions. It is also due to methodological choices adopted in LCA studies, including system boundaries (farm gate versus full-chain assessments) and allocation criteria. Consequently, lower values are typically observed for animals originating from dairy chains (particularly culled dairy cows), whereas higher impacts are associated with extensively managed herds or low-efficiency systems commonly found in lower-income countries [17,18].

Although two-thirds of the CFP is attributed to the farming phase alone, a full-chain quantification process should also account for the contribution of individual co- and by-products to the total footprint [1,18,19]. This is especially true considering that edible parts of a bovine represent on average ~50% of live weight, while the remaining part consists of hides, bones and other non-edible material. Literature reports that reusing non-edible material as such could create carbon credits able to offset the impact associated solely with processing. For this reason, it would be advisable to include this process in the LCA. However, most studies ignore the quantification of the impact of residual materials from processing, because they adopt a spatial boundary of the “from cradle to farm gate” type, focused on the main product only. This inevitably leads to an overestimation of emissions associated with meat alone.

One of the most important co-products of beef is the hide, which influences the value of overall by-/co-products by ~30–75% [20], and whose uses vary depending on animal category. The most valuable hides are those from calves and are used in the footwear and apparel industries. Hides from young bulls are used for automotive and furniture, while hides from adult animals are used to produce everyday items.

In the following paragraphs we delve into the partitioning of emissions generated in the farming and slaughter stages among the main bovine products (meat and milk) and the co-/by-products, including hides, which receive the main attention of this paper.

## 4. Carbon Footprint Allocation for Bovine Hides: From Slaughterhouse to Tannery

The question of whether hides should bear any environmental load prior to tanning would seem to have been settled by the United Nations Industrial Development Organization (UNIDO) [10], which, in assessing leather CFP, excludes farming and slaughter stages according to the following considerations:

“(a) *Raw hides must be considered co-products of renewable materials: a renewable resource is a natural resource with the capacity to reproduce itself through biological or natural processes and is replenished over time. Renewable resources are part of our natural environment and contribute to build the ecosystem. For cattle, sheep and goats, this definition perfectly applies to meat production (the determining product), which is a renewable material, with raw hides as co-products.*

(b) *Raw hides, the non-determining co-products, are not completely used, but at least partly substitute other products. As widely known in sector literature, a small part of the input raw materials (about 20–25%) is transformed into finished leather. The remainder consists of other by-products and animal wastes. At the same time, leather replaces other materials (mostly synthetics) in footwear, leather goods, clothing, automotive interiors and furniture.*

*Slaughtering is the intermediate process. Finished leather could also be credited for the avoided treatment of waste from raw hides entering the tanner. Therefore, system boundaries must be considered from the slaughterhouse, where activities and treatments to prepare hides for tanning (e.g., preservation through cooling or salting) end at the tannery gate.*”

In summary, according to UNIDO, since hides are waste subject to disposal that the leather industry valorises and recycles, they should reach the tannery with zero environmental footprint and, consequently, the CFP of leather products should be calculated only for tanning and industrial transformation.

Opposed to this, the scientific literature and environmental-audit opinions agree in assigning raw hides an environmental footprint, albeit a minimal one, depending on the objectives of the estimates and the allocation methods detailed below [21]; this also in view of concerns that international trade in these materials directly affects deforestation, as in analyses of commodity flows between Brazil and Italy [22].

The weak point in UNIDO’s guidelines is equating raw hides with waste, on the grounds that only 25% of input mass is effectively transformed into finished product and that, if not used by tanneries, hides would need disposal with associated costs. Against the first argument, it suffices to observe that all industrial processes produce offcuts, but their efficiency is tied to lower scrap per unit of finished product: buyers of raw materials are aware of disposal burdens and factor them into margin evaluations. Moreover, bovine raw hides are sold in Italy by slaughterers as food products precisely to enable trimming to be used by industries transforming them into gelatine or other products for human consumption [23]. Against the second argument, it suffices to note that when there is a positive market for raw hides (tanners pay slaughterers to acquire them, not vice versa for removal), hides must be considered co-products of bovine chains and therefore bear an environmental footprint. The question to address is thus the best allocation method to avoid unjustifiably inflating (or deflating) the footprint of raw hides. In the next paragraphs we detail allocation, provide a brief literature analysis for beef chains and report original examples from large-scale Italian experience. Although the GWP* metric provides a more accurate representation of the climate impact of short-lived climate pollutants such as methane, it was not applied here because the available emission data are expressed as annual CO_2_*e* under GWP_100_, and conversion to GWP* would require detailed temporal emission profiles (herd dynamics and methane flow data) that are unavailable at the required resolution. The discussion therefore refers to GWP_100_ values, as in current LCA and policy frameworks.

## 5. The Problem of Environmental Impacts Allocation in Bovine Chains

Among the many procedures proposed for correct partition between milk and meat, the following represent the most important:

(a) bio-physical methods, preferred by researchers as they are based on partitioning physical and energy flows of the system among products;
(b) physical methods, preferred by production chains as they allow allocation of explicit and hidden values and costs on a weighted basis;
(c) economic methods, preferred by LCA professionals as they are simpler with respect to concrete values to be recognised [16].

The differences between bio-physical and physical allocation methods are generally limited because both ultimately reflect animal physiology and carcass composition. Bio-physical methods allocate impacts based on metabolizable energy used for tissue growth or milk production, whereas physical (mass-based) methods distribute impacts according to the relative weight of outputs. However, the extent of differences between bio-physical and physical allocation can vary depending on the system boundaries, co-product definitions, and data used, as highlighted by Wilfart et al. [16], who showed that while results are often similar, larger divergences may occur in certain meat production systems. These methodological options do not correspond to different professional roles. References to (a), (b) and (c) merely demonstrate the variety of allocation criteria employed in LCA studies and do not suggest that researchers and practitioners fall into distinct categories. Rather, they highlight the coexistence of scientifically grounded, production-oriented, and market-based approaches to allocating environmental burdens.

In the case of dairy cattle CFP, the IDF [24] suggests a standard bio-physical split of 85% to milk and 15% to meat (total sold liveweight), but Ineichen et al. [25] showed that bio-physical assignment is proportional to herd milk yield, varying from over 20% for <2 kL production to under 5% for >8 kL production. Kyttä et al. [21] in their literature review and expert auditor survey found that bio-physical partition, assessed with different methods, is close to the IDF value, but farmers’ perceived intrinsic value assigned to individual products and co-products diverges, placing 50–60% on animals and the remainder on milk.

Allocation becomes more complex when assessing the weight of the various parts of animals destined for slaughter, whether from dairy or beef systems. Cattle are part of an articulated circular system that can supply, besides meat, a whole series of co- and by-products generated during slaughter [26]. As mentioned above, raw hides are included into the co-product category, having value and thus an active market.

At present, there is no consensus among authors on the allocation method to quantify the environmental impact of by-/co-products remaining after beef production and generating economic value at stages following farming. However, literature analysis and auditor opinions suggest that economic allocation is the most popular method used in beef (and other species) supply chains [16]. The method chosen inevitably influences the impact of individual outputs; nonetheless, re-use of beef co-/by-products will be increasingly important in the transition to a circular economy and, therefore, it is appropriate to recognise them as values generated by primary production and to detail a specific methodology that includes them in LCAs of bovine-chain products and services.

In light of the above, three practical conclusions can be drawn, each of which is firmly rooted in the evidence discussed in this section. Firstly, raw hides must be considered co-products because they have a positive and independent market value, meaning they cannot be considered as waste. This aligns with the definition of co-products used in LCA practice and the economic evidence reported above. Secondly, carbon-impact evaluation should extend at least to the slaughterhouse gate, since hides undergo preservation, handling, and valorisation processes that render them marketable outputs. Excluding these steps would omit part of the upstream burden associated with preparing them for the leather production process. Thirdly, the most appropriate method for allocating impacts to hides is economic allocation, since their value is not causally linked to animal physiology or tissue growth (as with bio-physical or mass-based allocation), but rather to their market price. This approach is also the one most widely recommended in international LCA guidance for bovine chains [14,21]. Together, these elements ensure transparent and coherent treatment of hides in cattle LCAs.

## 6. Allocation of the Environmental Impacts in the Beef Chain and the Weight of Hides

According to Kyttä et al. [21], considering an economic allocation for the beef chains, it results that the 87% of the impact is associated with meat and the 13% with co-/by-products; of the later, the hide accounts for just under 5% of the total. The economic approach better represents demand-supply environmental consequences but makes temporal comparisons harder [20]. Compared to the economic allocation, the bio-physical allocation, based on partitioning metabolizable energy used by the animal for growth of different tissues separated at slaughter [27], allocates 80% of the impact to meat and 20% to co-/by-products. Meat’s impact decreases further with a mass (physical) allocation (73%), while that of co-/by-products increases (27%); in this case, note that the hide’s share of total live weight varies by breed and sex [28]. Hide share of live weight tends to be greater in specialised beef breeds than in dairy breeds, and in tropical-origin breeds than in European ones [20]. Table 2 reports physical allocation for different animal categories, arising from a large sample within the INALCA system (Cremonini Group). Physical allocation of hide ranges from a minimum of 4.2% in cull dairy cows (27 kg hide on 647 kg live weight at slaughter) to a maximum of 6.9% in semi-heavy young bulls (37 kg hide on 538 kg liveweight), with a mean of 5.88% and an average slaughter liveweight of 555 kg.

Economic allocation of hide, for the same extensive sample (Table 3), shows markedly lower values for all animal categories, at less than half those calculated by physical allocation, averaging 2.68% for 2023.

However, these values, if recalculated over the historical series in Table 4, tend to fall due to the relative loss of hide value compared with meat: for dairy cows, for example, the ratio was 0.70 in 2015, falling to 0.19 in 2023. As discussed at length in the LCA literature, economic allocation is sensitive to temporal price variability, and annual fluctuations in hide and meat values can significantly alter allocation shares. Several authors therefore recommend recalculating economic allocation on a yearly basis and applying multi-year averages when long-term comparability is required to smooth out short-term volatility [16,21]. This approach is also consistent with the FAO LEAP Guidelines [29], which explicitly recommend using five-year averages of economic values for slaughter co-products to obtain stable and comparable allocation factors over time. Compared with literature, it should be noted that the INALCA survey separated only hides and fat from the remainder of carcass products and co-products. The “meat” category here includes edible fifth-quarter parts; as a result, values reported in Table 2 and Table 3 for the main product are considerably higher than those drawn from the literature.

Taken together, these comparisons show that economic allocation is the most suitable approach for hides. Unlike bio-physical and mass-based methods, which are driven by criteria that are unrelated to hide value, such as physiology or structure, economic allocation reflects the actual market relevance of hides representing also the approach most widely recommended in international LCA guidance for bovine chains. It prevents too the footprint of meat from being artificially inflated by ensuring that all marketable outputs bear a proportional share of the upstream burden. For these reasons, economic allocation provides the most transparent and policy-aligned basis for accounting for the upstream climate impacts of raw hides.

## 7. Estimating the Climate Impacts of Raw Bovine Hide

The impact associated with hide production can be expressed per unit mass or surface. For raw hides, the kilogram represents the common FU, while for finished leather it is represented by the square metre [30]. The CFP per kg of raw hides in the only reference found in the literature is 12.3 kg CO_2_*e* with economic allocation, rising to 12.9 kg CO_2_*e* with mass allocation [20].

The INALCA group data (2024) [11] on overall climate impacts allow calculation of the CFP per kg of raw hide, also accounting for the slaughterhouse perimeter, equal to 3.55 kg CO_2_*e* under physical allocation and 1.63 kg CO_2_*e* under economic allocation for 2023.

Pignatelli and Marino [31] evaluated the impact of 1 kg of raw hide at the farm gate as 1.9 kg CO_2_*e* for young bulls and 0.97 kg CO_2_*e* for dairy cows, without specifying the allocation method used. Table 5 reports the data of Vicenza tanning district [32], calculated as m^2^ of processed leather, on minimum, maximum and average yields, based on the INALCA’s CFP per kg raw hide.

The values obtained are highly variable as a function of raw-hide-to-surface yield, but an average upstream CFP (farming and slaughter) of ~13.5 kg CO_2_ per m^2^ is reasonable in relation to finished-product data reported below. These differences primarily reflect variations in hide thickness, quality grade, and usable surface area among different animal categories. Animals with larger bodies or thicker hides (e.g., adult beef cattle) generate more square metres of usable surface area per hide, thereby reducing the impact per m^2^ further upstream in the supply chain. Conversely, hides from dairy cows or younger animals tend to have a smaller usable area due to thinner grain, defects or an irregular shape. Additionally, the effective yield depends heavily on the intended use of the leather. Coarse goods manufacturing (e.g., upholstery and heavy leather goods) can utilise a larger proportion of the hide, whereas high-end fashion applications select only the finest central portions and discard the belly, neck, and scarred areas. This selective use substantially reduces the usable surface area for premium products, leading to higher climate impact values per m^2^. Together, these factors explain the wide variation observed in Table 5.

A recent assessment for a tannery estimated upstream farming-and-slaughter impact at 6.78 kg CO_2_*e* per m^2^ of processed leather [33]. For finished leather, the literature CFP ranges between 64.8 and 151.9 kg CO_2_*e*/m^2^ [30,34]. Among finished leathers, aniline leather is particularly valuable as processing confers a “natural effect” [35]: the CFP of aniline leather increases with thickness, and 78% of the impact is attributed to extraction of raw materials needed for processing, with the remainder from internal manufacturing energy (21%) and distribution (<1%) [30].

Ultimately, the CFP of hides, analogously to meat, must also consider the tanning system’s effective capacity to generate co-products such as splits for collagen production, so as to reward operators who achieve such recoveries compared with those taking non-food hides and thus unable to generate co-products for the food or feed sectors.

## 8. Limitations of the Study

In order to ensure clear interpretation of the findings, it is important to highlight the limitations of this work. Firstly, while the GWP* metric would offer a more accurate outcomes of the warming impact of short-lived climate pollutants (e.g.,: methane), it could not be used due to the lack of the detailed temporal emission data (herd dynamics and methane flow series), that are necessary for its application. In addition, all results were expressed in terms of GWP_100_ to maintain consistency with existing inventories and current LCA standards.

Secondly, the empirical evidence supporting the allocation estimates arise from large-scale Italian data. While these data are extensive, highly detailed, and representative of one of the most widespread European beef supply chains, their numerical values may not be directly generalizable to production systems operating under different structural, economic, or climatic conditions. Although the methodological considerations remain broadly applicable, specific allocation shares should therefore be interpreted in light of the context from which they were generated.

Thirdly, although economic allocation is widely used in LCA practice and conceptually aligned with market valuation, it is intrinsically sensitive to price variability. Hide-to-meat price ratios fluctuate over time and this can impact allocation shares from one year to the next. To mitigate this limitation, we recommend multi-year averaging when economic allocation is used for comparative or regulatory purposes. This approach provides more stable and comparable allocation results over time.

Finally, the industrial data available for this study separated hides and fat from the remaining co-products, which were aggregated into a single “meat” category. While this reflects data-reporting practices in the slaughter industry, it may affect comparability with studies that adopt more granular classifications of co- and by-products [36].

## 9. Conclusions

Attributing climate-altering impacts to leather products deriving from raw hides from the farming and slaughter stages (so-called upstream) is debated and controversial across the supply chain. On the one hand, the tanning industry claims its role as valoriser of a product otherwise destined for disposal; on the other, farmers and slaughterers think correct to load a share, albeit minimal, of emissions also onto raw hides.

Scientific literature agrees on allocating an emission share to slaughter co-products, as these are secondary parts of the animal, subject to market exchange. However, there is debate on how to subdivide it: bio-physical methods, considered the most scientifically correct, are complex to adopt and impose excessive loads on co-products; physical methods share this drawback without the precision advantage; economic methods, although affected by temporal and local price variability, are nevertheless considered best as they aim to attribute to the main and co-products a climate burden proportional to market value and to avoid green-washing of the main product at the expense of secondary ones usually hidden from the consumer’s attention.

The results obtained in this study confirm H1, showing that raw hides, being co-products with a positive market value, bear a non-zero share of upstream emissions; attributing them a null footprint would introduce systematic bias. They also confirm H2, as economic allocation consistently assigns a lower burden to hides than physical (mass-based) allocation across animal categories, in line with price-based valuation and LCA practice. Evidence also supports H3, since the hide share under economic allocation varies over time with the hide/meat price ratio, and multi-year averaging provides more stable values for LCA comparability. None of the hypotheses is confuted by the results presented here.

Finally, the tanning and leather goods industry should calculate the incoming emission load of raw hides by requiring suppliers to provide a transparent LCA for this raw material using economic allocation. However, the lower the final upstream burden per unit product (m^2^ of processed leather), the greater the operator’s ability to add value to raw hides in physical (or economic) terms, and to conveniently redirect processing offcuts to the collagen and hydrolysed protein industries. The GWP* was considered but not applied due to the lack of the temporal herd emission series necessary for its correct implementation. Nonetheless, the conclusions remain robust under the standard GWP_100_ accounting framework and are aligned with current international LCA guidance.

In the coming years, more comprehensive evaluations could incorporate hide allocation into full-chain modelling of the leather manufacturing process under both GWP_100_ and GWP*, provided that suitable temporal emission data is available. Improving transparency at the intersection of the cattle and leather industries will also help to align environmental accounting methods with the practical needs of both sectors.

## Figures and Tables

**Table 2:** Physical allocation of raw hide in the INALCA system.

Category *	Liveweight (kg)	Hide Weight (€/kg)	Hide Allocation (% of Live Weight)
Young bulls	538	37	6.9%
Calves	346	23	6.6%
Young bulls and heifers	618	38	6.1%
Culled dairy cows	627	35	5.6%
Culled dairy cows	647	27	4.2%

* Data from different slaughterhouses (about 750,000 slaughtering/y).

**Table 3:** Economic allocation of raw hide in the INALCA system (average prices in 2023).

Item *	Young Bulls		Calves		Young Bulls and Heifer		Culled Dairy Cows		Culled Dairy Cows	
Meat	1260€	95.6%	899€	92.5%	1997€	96.9%	1600€	97.0%	992€	96.0%
Fat	21€	1.6%	41€	4.2%	17€	0.8%	6€	0.4%	15€	1.5%
Hide	37€	2.8%	32€	3.3%	46€	2.2%	43€	2.6%	26€	2.5%
TOTAL	1318€	100%	972€	100%	2060€	100%	1649€	100%	1033€	100%

* Data from different slaughterhouses (about 750,000 slaughtering/y).

**Table 4:** Wholesale price trends (€/kg) in Italy for beef products and co-products (INALCA data).

Animal Part	2023	2022	2021	2020	2019	2018	2017	2016	2015
Animal fat	1.16	1.55	1.11	0.82	0.71	0.68	0.80	0.72	0.67
Hides	1.24	1.29	1.23	1.14	1.47	1.95	2.33	2.25	2.04
Cow meat	6.39	6.16	4.28	3.98	4.21	3.82	3.52	4.13	3.41
Young-bull meat	6.40	6.34	5.07	4.86	4.90	4.90	4.84	4.86	4.77
Veal	6.69	6.75	6.90	6.12	6.21	6.41	6.27	5.68	5.59

**Table 5:** Carbon footprint per m^2^ of leather processed in the Vicenza district (calculated on the INALCA value of carbon footprint).

Allocation	Minimum (m^2^/kg)	Minimum (CO_2_/m^2^)	Maximum (m^2^/kg)	Maximum (CO_2_/m^2^)	Average (m^2^/kg)	Average (CO_2_/m^2^)
Physical	0.09	39.44	1.57	2.26	0.12	29.58
Economic	0.09	18.11	1.57	1.04	0.12	13.58

## Data Availability

Data will be made available on request.

## Abbreviations

The following abbreviations are used in this manuscript:

CFP	Carbon Footprint
GHG	Greenhouse Gas
GTP	Global Temperature Potential
GWP	Global Warming Potential
GWP*	Global Warming Potential star
FU	Functional unit
IDF	International Dairy Federation
LCA	Life Cycle Assessment
LULUCF	Land Use, Land Use Change and Forestry
